# 2:1 Multiplexing Function in a Simple Molecular System

**DOI:** 10.3390/s120404421

**Published:** 2012-03-30

**Authors:** Sha Xu, Yu-Xin Hao, Wei Sun, Chen-Jie Fang, Xing Lu, Min-Na Li, Ming Zhao, Shi-Qi Peng, Chun-Hua Yan

**Affiliations:** 1 School of Chemical Biology and Pharmaceutical Sciences, Capital Medical University, Beijing 100069, China; E-Mails: xusha0810@126.com (S.X.); haoyuxin0829@163.com (Y.-X.H.); xing_lu@yeah.net (X.L.); minnali78@126.com (M.-N.L.); 2 Beijing National Laboratory for Molecular Sciences, State Key Lab of Rare Earth Materials Chemistry and Applications & PKU-HKU Joint Lab in Rare Earth Materials and Bioinorganic Chemistry, Peking University, Beijing 100871, China; E-Mail: sunweidavid1@gmail.com

**Keywords:** fluorescence, anthracene, molecular 2:1 multiplexer

## Abstract

1-[(Anthracen-9-yl)methylene] thiosemicarbazide shows weak fluorescence due to a photo-induced electron transfer (PET) process from the thiosemicarbazide moiety to the excited anthracene. The anthracene emission can be recovered via protonation of the amine as the protonated aminomethylene as an electron-withdrawing group that suppresses the PET process. Similarly, chelation between the ligand and the metal ions can also suppress the PET process and results in a fluorescence enhancement (CHEF). When solvents are introduced as the third control, a molecular 2:1 multiplexer is constructed to report selectively the inputs. Therefore, a molecular 2:1 multiplexer is realized in a simple molecular system.

## 1. Introduction

There is increasing interesting in exploring single molecule species that could be potentially applied in the construction of binary logic devices and future computers at the molecular scale [1–8]. Since the first molecular AND gate was reported [9], all common essential logic gates including AND, NOT, OR, YES, INHIBIT, XOR, NAND and NOR, which are used in conventional silicon circuitry, have been mimicked at the molecular level with chemical or optical signals [10–21]. With all these logic gates in hand, the next step is to construct molecular logic networks taking advantage of functional integration within a single molecule via rational chemical design. This is prior to relying on extensive physical connection of elementary gates. Recently, molecular-scale arithmetic has also been reported [21–30]. Those single molecule-based combinatorial circuits are more important because they are fundamental to a complex information processing system. Tian reported a fluorophore capable of logic memory [31]. We have systematically explored the combination of logic functions and realized a safe computing platform with user-identity-directed arithmetic functions to defend information risk [32,33]. An important function in information technology, e.g. signal multiplexing, has been realized based on the molecules [34–36]. The construction of molecule-based 1:2 digital demultiplexer was also reported [37,38]. The signal multiplexing/demultiplexing was demonstrated in 8-methoxyquinoline and enzymes [39,40]. In spite of various logic functions mimicked at molecular level, however, the combination and integration of advanced functions is still in the infant stage and reports on this subject are rare [41–45].

In our previous work, up to seven binary logic gates were realized within a single molecule, in which redox-active tetrathiafulvalene (TTF) was utilized as a switch to control the fluorescence [46]. Herein, we report a molecular system which is capable of performing multiplexing function in response to chemical stimuli.

**Figure 1.**
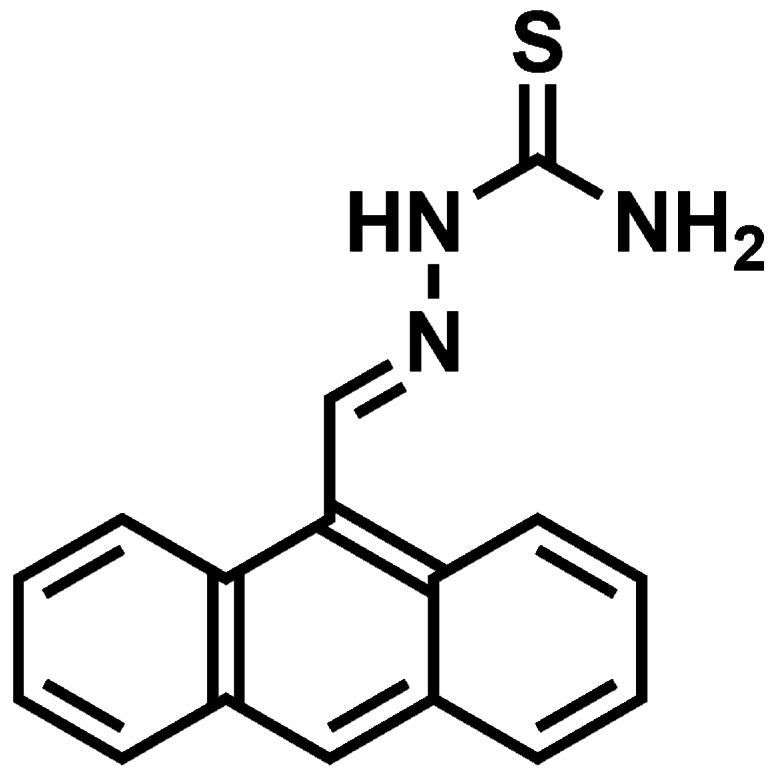
The molecular structure of the ligand L.

In the present work, the fluorescence of anthracene in the simple molecule 1-[(anthracen-9-yl) methylene] thiosemicarbazide (L, Figure 1) [47,48] is tuned to realize a molecular 2:1 multiplexer with anthracene as a signal unit via tuning the PET process. Protonation of amine and chelation with metal ions can suppress the photo-induced electron transfer (PET) process. Combined with the solvents as control inputs to switch the fluorescent output, the ligand L can report the binary state of either one of these inputs or the other.

## 2. Experimental Section

The UV-vis absorption spectra were recorded on a Shimadzu 2500 UV-VIS spectrophotometer. The fluorescence spectra were recorded on a Shimadzu RF-5301 spectrofluorophotometer using 5 nm input and 5 nm output width. ^1^H- and ^13^C-NMR (TMS) are recorded on a Bruker Avance II 500MHz spectrometer. The mass spectra were measured on a Waters Quattro micro TM API mass spectrometer. Elemental analyses were performed on a Vario EI Elementar system.

## 3. Results and Discussion

### 3.1. Synthesis of the Ligand L

When anthracene-10-carbaldehyde was treated with thiosemicarbazide in methanol, the ligand L was readily formed as an orange microcrystalline solid appearing in the reaction medium, and it was characterized with elemental analysis (EA), IR, MS, and NMR spectra data. ^1^H-NMR (DMSO-*d*_6_) δ = 7.58 (m, 2H, anthryl), 7.65 (m, 2H, anthryl), 7.73 (s,1H, NH_2_), 8.15 (d, 2H, anthryl), 8.32 (s, 1H, NH_2_), 8.58 (d, 2H, anthryl), 8.71 (s, 1H,CH=N), 9.34 (s, 1H, anthryl), 11.66 (s, 1H, NH). ^13^C-NMR (DMSO- *d*_6_) δ = 125.2, 125.5, 126.0, 127.8, 129.4, 129.9, 130.1, 131.4, 142.7, 178.6. IR (KBr): ν = 3436 (NH_2_), 3250 (NH), 1601 (C=N), 1285 (C=S) cm^−1^. ESI-MS, m/z (%): 279 [M^+^]. EA (%): C_16_H_13_N_3_S, calcd.: N, 15.05; C, 68.82; H, 4.66; found: N, 15.25; C, 68.61; H, 4.80.

### 3.2. Photophysical Properties of the Ligand L

**Figure 2.**
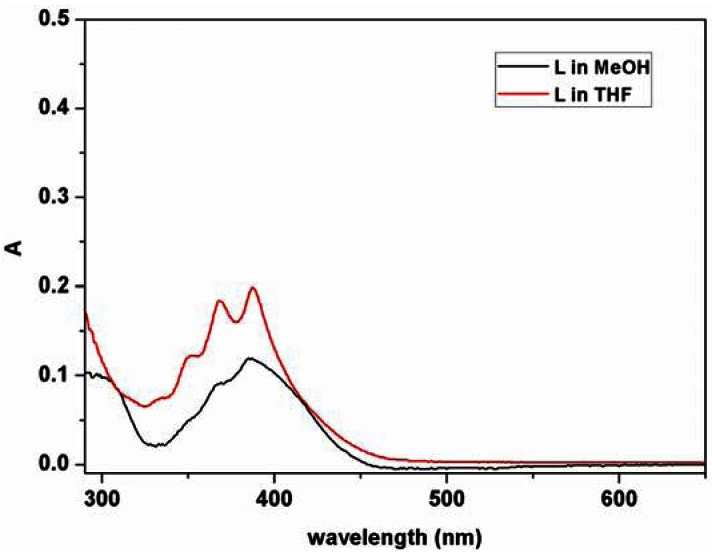
Absorption spectra of the ligand L in THF and methanol (2.0 × 10^−5^ M).

The photophysical properties of the ligand L were initially examined in tetrahydrofuran (THF) and methanol. As shown in Figure 2, characteristic anthracene absorption bands appear in the range of 335–500 nm in UV-Vis spectra [47].

**Figure 3.**
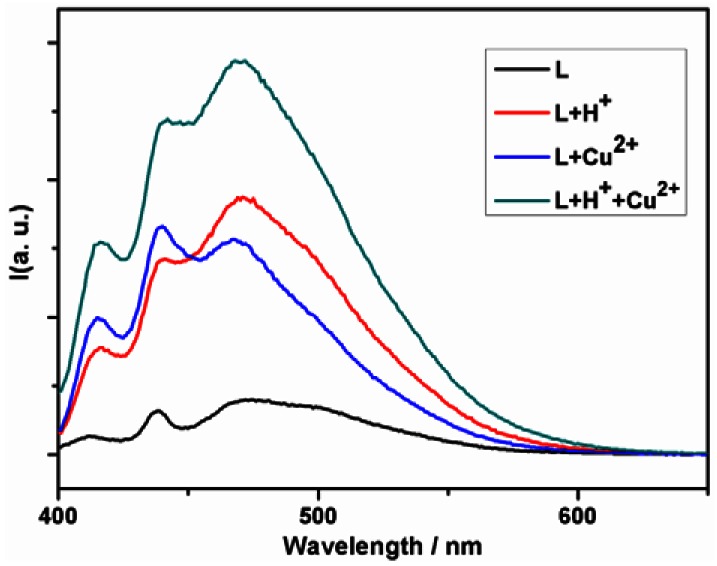
Fluorescence emission spectra of the ligand L with addition of H^+^ and/or Cu^2+^ in THF (2.0 × 10^−5^ M).

The fluorescence spectrum of the ligand in THF solution displays peaks at 412, 437 and 470 nm (Figure 3). The emission intensity of the ligand L is significantly reduced through a photo-induced electron transfer (PET) process, with the electron-transfer from the electron-donor thiosemicarbazide moiety to the excited anthracene. When the amine group is proton-free, it could serve as a PET donor (ΔG_PET_ = −0.1 eV) [49–51]. If protons are present in sufficient concentration, the protonated aminomethylene behaves as an electron-withdrawing group which disturbs and suppresses the PET process from the thiosemicarbazide moiety, thus producing an enhancement of the fluorescent emission. In addition, chelation enhanced fluorescence (CHEF) is popular for chemosenors base on PET mechanism to sensing metal ions [52]. Two above points have been useful to design chemosensors based on the PET mechanism.

**Figure 4.**
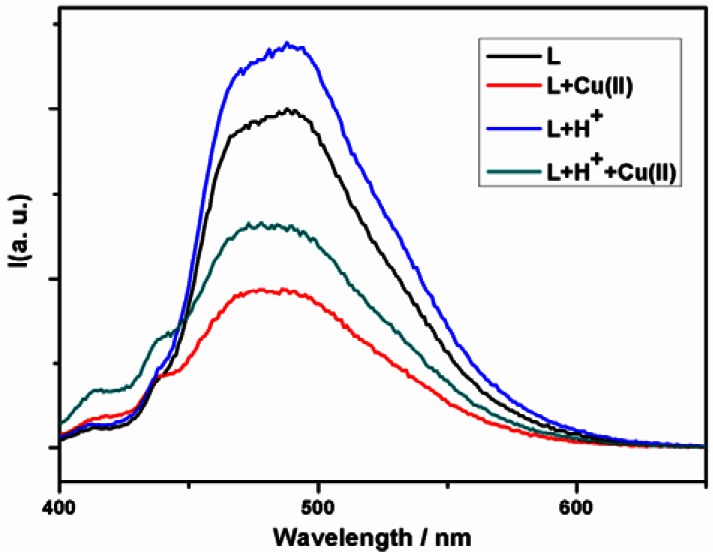
Fluorescence emission spectra of the ligand L with addition of H^+^ and/or Cu^2+^ in methanol (2.0 × 10^−5^ M).

Solvents also impact the fluorescent properties of the ligand. The ligand L exhibits a weak emission band centered at 470 nm in THF (Figure 3) whereas it exhibits a relatively stronger emission centered at 481 nm in methanol (Figure 4), with the vibration fine structure missed and the fluorescence maximum wavelength red-shift. This phenomenon induced by polar solvents has been reported, and it has been attributed to the formation of exciplexes or specific solute-solvent complexes in the excited state [53].

**Figure 5.**
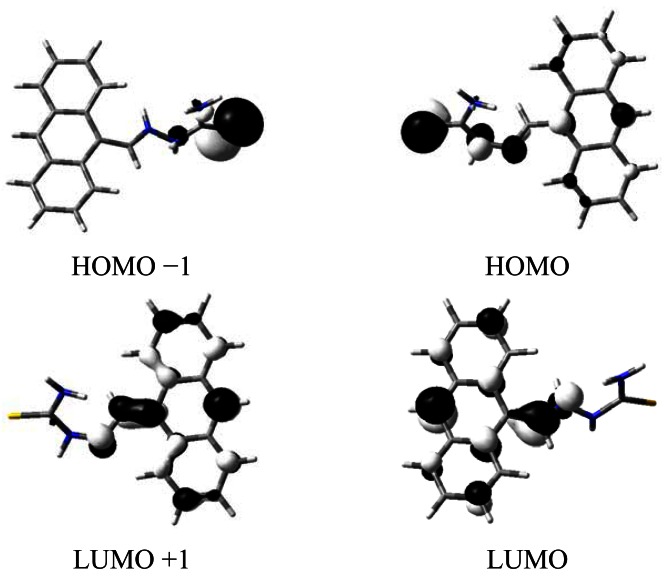
HOMO −1, HOMO, LUMO and LUMO +1 distributions of the ligand at the B3LYP/6-31G (d,p) level of theory calculated.

The emission enhancement is significant under the addition of acid to the THF solution of the ligand, while the emission is slightly enhanced in the methanol solution. The enhanced fluorescence upon protonation at amine group is caused by the sufficient protons which disturb and suppress the PET process and result in a recovered emission [12,32,33]. The similar enhancement due to CHEF effect is observed upon addition of copper ions to the THF solution. The ESI-MS result of the mixture of the ligand and copper ions show a *m/z* peak at 297.1 ([M^+^+H_2_O]) and 659.2 ([CuL_2_(H_2_O)_2_]). To understand the chelation ability of the ligand towards Cu^2+^, the HOMO (high occupied molecular orbit) and LUMO (lowest unoccupied molecular orbit) distribution of the L were determined by density functional theory (DFT) calculations. As shown in Figure 5, the HOMO distribution is mainly located diffusely over the thiourea group, thus the S and N atoms are electron-rich centers and exhibit high affinity for the metal ion, which contributes to CHEF effect.

In methanol solution, however, the fluorescence is quenched. This might be due to the intrinsic fluorescence quenching by mechanisms inherent to paramagnetic species Cu^2+^ in high dielectric and polar methanol. The communication between paramagnetic Cu^2+^ and fluorophore is predominant, and thus the fluorescence is quenched.

### 3.3. Binary Logic Analysis for Molecular Logic Circuit

Fluorescence is one of the most widely employed signals owing to its high sensitivity, feasibility in detection, and low cost in operation. Since systems containing fluorophores can be switched between emissive state and quenched state via manipulation of the PET process, the chemical system described above could be a simple functional model of logic gate with tuning the fluorescence as output signal.

It is interesting to note that the logic analysis of the present system is complex if the solvent is considered as the control input. In this context, the logic system described here is a chemical model of a 2:1 multiplexer. Physically, a multiplexer is a communication device that combines multiple inputs into an aggregate signal to be transported via a single transmission channel. Simply, it is a data selector which outputs any one of several possible inputs via a control switch.

**Figure 6.**
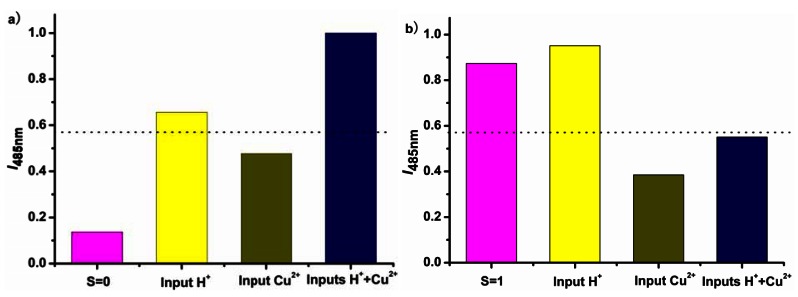
The fluorescent emissive intensities of the ligand L at 485 nm with excitation at 387 nm, the changes of which mimic a digital 2:1 multiplexer at molecular level. The ligand was dissolved in THF and methanol under a concentration of 2.0 × 10^−5^ M, into the solutions HCl was added at two equivalent ratio and one equivalent CuCl_2_ was applied. The use of the solvent is denoted as the control switch, with (**a**) the use of THF as control S off and (**b**) use of methanol as control S on. The dotted line signifies the threshold level for an on response of the fluorescence output.

In the present case, the solvent as the third input acts like a mechanical rotary switch to control the logic functions of the molecule, enabling the molecule to output selectively the desirable data from all the inputs. With the input of the proton and the metal ion Cu^2+^ while solvent as the control, changes of the fluorescent emission of the ligand corresponding to two different inputs mimic the digital selection function (Figure 6). In THF solution, the weak fluorescent state is set as the initial state of the logic 0, since the emission intensity monitored at 485 nm is below the threshold. The protonation enhances the fluorescence with the emission intensity beyond the threshold, which is denoted as output 1. The fluorescence is slightly enhanced with introduction of Cu^2+^, however, the intensity is still below the threshold and thus it is denoted as output 0. When both proton and Cu^2+^ are added into the system simultaneously, the fluorescent emission is remarkably enhanced, due to the coordination of the metal ions with the protonated ligands. It means in THF the ligand responses to the input H^+^.

**Figure 7.**
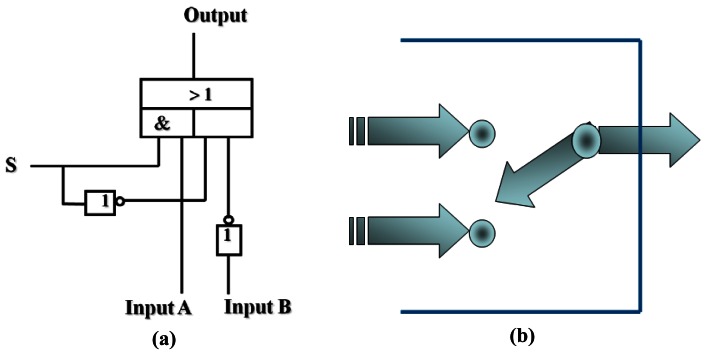
(**a**) The equivalent circuit; (**b**) A scheme of a multiplexer.

In digital design of a multiplexer, where both the inputs and outputs are voltages, the switch for the control is usually an analogous of the mechanical rotator. However, in most molecular logic gates and devices, chemical inputs and photonic outputs are applied, and it is reasonable for the chemical model of molecular devices to choose the solvents used as control switch. If the use of solvent THF is set as the Off state, corresponding to the Off state of a mechanical switch, and the use of MeOH solvent is corresponding to the switch On state, the resulting output of the present system is in agreement with the selective response to any functions of the input sets. In MeOH solution, Cu^2+^ quenches the emission of the ligand L. Although the fluorescence is enhanced with the proton added, it is quenched and the intensity is below the threshold when the protons and Cu^2+^ are both introduced, indicating that the system is selectively responsive to Cu^2+^ even in the presence of the proton. For the truth table calculation, the emission results in MeOH could be interpreted by using the negative logic rule. Combined with the fluorescent response in both MeOH and THF solvents, the chemical system is capable of mimicking a 2:1 multiplexer function with the solvent switch (Figure 7, Table 2).

## 4. Conclusions

In summary, we have demonstrated a combinational logic circuit of molecular multiplexer within a simple molecular system, where the emission of anthracene and the solvent effect are employed as signaling and switch control, respectively. The work presented herein opens the possibility for further development of chemical logic systems, and thus for the construction of molecular digital devices which are likely to be evolved into components of future molecular computers such as a wet computer. Further investigation of this topic is underway in our lab.

## Figures and Tables

**Table 2.** The truth table for the molecular 2:1 multiplexer.

**Input A** **H^+^**	**Input B** **Cu^2+^**	**Selection** **Control S** ^a^	**Output** ^b^ **FL**
0	0	0	0
0	1	0	0
1	0	0	1
1	1	0	1
0	0	1	0
0	1	1	1
1	0	1	0
1	1	1	1

a The use of solvent is set as control S with THF as 0 and MeOH as 1;

b The output in MeOH is interpreted by using the negative logic convention.
